# Mutual Information Rate and Bounds for It

**DOI:** 10.1371/journal.pone.0046745

**Published:** 2012-10-24

**Authors:** Murilo S. Baptista, Rero M. Rubinger, Emilson R. Viana, José C. Sartorelli, Ulrich Parlitz, Celso Grebogi

**Affiliations:** 1 Institute for Complex Systems and Mathematical Biology, Scottish Universities Physics Alliance, University of Aberdeen, Aberdeen, United Kingdom; 2 Institute of Physics and Chemistry, Federal University of Itajubá, Itajubá, Brazil; 3 Instituto de Ciências Exatas, Departamento de F sica, Universidade Federal de Minas Gerais, Belo Horizonte, Brazil; 4 Institute of Physics, University of São Paulo, São Paulo, Brazil; 5 Biomedical Physics Group, Max Planck Institute for Dynamics and Self-Organization, Göttingen, Germany; 6 Institute for Nonlinear Dynamics, Georg-August-Universität Göttingen, Göttingen, Germany; 7 Freiburg Institute for Advanced Studies, University of Freiburg, Freiburg, Germany; Universidad de Zarazoga, Spain

## Abstract

The amount of information exchanged per unit of time between two nodes in a dynamical network or between two data sets is a powerful concept for analysing complex systems. This quantity, known as the mutual information rate (MIR), is calculated from the mutual information, which is rigorously defined only for random systems. Moreover, the definition of mutual information is based on probabilities of significant events. This work offers a simple alternative way to calculate the MIR in dynamical (deterministic) networks or between two time series (not fully deterministic), and to calculate its upper and lower bounds without having to calculate probabilities, but rather in terms of well known and well defined quantities in dynamical systems. As possible applications of our bounds, we study the relationship between synchronisation and the exchange of information in a system of two coupled maps and in experimental networks of coupled oscillators.

## Introduction

Shannon’s entropy quantifies information [1]. It measures how much uncertainty an observer has about an event being produced by a random system. Another important concept in the theory of information is the mutual information [1]. It measures how much uncertainty an observer has about an event in a random system **X** after observing an event in another random system **Y** (or vice-versa).

Mutual information (MI) is an important quantity because it quantifies not only linear and non-linear interdependencies between two systems or data sets, but also is a measure of how much information two systems exchange or two data sets share. Due to these characteristics, it became a fundamental quantity to understand the development and function of the brain [2], [3], to characterise [4], [5] and model complex systems [6]–[8] or chaotic systems, and to quantify the information capacity of a communication system [9]. When constructing a model of a complex system, the first step is to understand which are the most relevant variables to describe its behaviour. Mutual information provides a way to identify those variables [10].

However, the calculation of mutual information in dynamical networks or data sets faces three main difficulties[4], [11]–[13]. Mutual information is rigorously defined for random memoryless processes, only. In addition, its calculation involves probabilities of significant events and a suitable space where probability is calculated. The events need to be significant in the sense that they contain as much information about the system as possible. But, defining significant events, for example the fact that a variable has a value within some particular interval, is a difficult task because the interval that provides significant events is not always known. Finally, data sets have finite size. Probabilities computed from finite data sets are subjected to unavoidable sampling errors. As a consequence, mutual information can often be calculated with a bias, only [4], [11]–[13].

In this work, we show how to calculate the amount of information exchanged per unit of time [Eq. (2)], the so called mutual information rate (MIR), between two arbitrary nodes (or group of nodes) in a dynamical network or between two data sets. Each node represents a d-dimensional dynamical system with 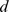 state variables. The trajectory of the network considering all the nodes in the full phase space is denoted by 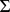 and represents the “attractor”, which in the following calculations is considered to be an asymptotic limiting set. Then, we propose an alternative method, similar to the ones proposed in Refs. [14], [15], to calculate significant upper and lower bounds for the MIR in dynamical networks or between two data sets, in terms of Lyapunov exponents, expansion rates, and capacity dimension. These quantities can be calculated without the use of probabilistic measures. As possible applications of our bounds calculation, we describe the relationship between synchronisation and the exchange of information in small experimental networks of coupled Double-Scroll circuits.

In previous works of Refs. [14], [15], we have proposed an upper bound for the MIR in terms of the positive Lyapunov exponents of the synchronisation manifold. As a consequence, this upper bound could only be calculated in special complex networks that allow the existence of complete synchronisation. In the present work, the proposed upper bound can be calculated to any system (complex networks and data sets) that admits the calculation of Lyapunov exponents.

We assume that an observer can measure only one scalar time series for each one of two chosen nodes. These two time series are denoted by 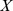 and 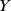 and they form a bidimensional set 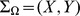, a projection of the “attractor” into a bidimensional space denoted by 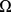. To calculate the MIR in higher-dimensional projections 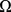, see Information S1. To estimate the upper bound of the MIR in terms of Lyapunov exponents obtained from the reconstructed attractor of a scalar time-series see Information S1. Assume that the space 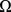 is coarse-grained in a square grid of 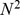 boxes with equal sides 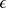, so 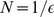.

Mutual information is defined in the following way [1]. Given two discrete random variables, **X** and **Y**, each one produces events 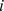 and 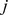 with probabilities 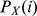 and 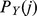, respectively, the joint probability between these events is represented by 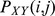. Then, mutual information is defined as
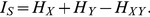
(1)



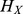
  =  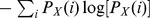, 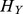  =  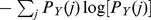, and 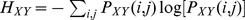. When using Eq. (1) to calculate the mutual information between the dynamical variables 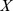 and 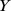, the probabilities appearing in Eq. (1) are defined such that 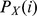 is the probability of finding points in a column 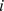 of the grid, 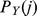 of finding points in the row 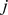 of the grid, and 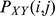 the probability of finding points in a box where the column 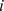 meets the row 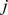 of the grid.

The MIR was firstly introduced by Shannon [1] as a “rate of actual transmission” [16] and later more rigorously redefined in Refs. [17], [18]. It represents the mutual information exchanged between two dynamical variables (correlated) per unit of time. To calculate the MIR, the two continuous dynamical variables are transformed into two discrete symbolic sequences 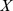 and 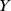. Then, the MIR is defined by
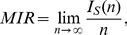
(2)where 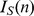 represents the usual mutual information between the two sequences 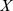 and 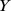, calculated by considering words of length 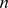. If 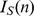 is calculated using 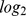, the MIR in Eq. (2) has units of bits/symbol. If a discrete system is producing the symbols, the units of Eq. (2) are bits/iteration.

The MIR is a fundamental quantity in science. Its maximal value gives the information capacity between any two sources of information (no need for stationarity, statistical stability, memoryless) [19]. Therefore, alternative approaches for its calculation or for the calculation of bounds of it are of vital relevance. Due to the limit to infinity in Eq. (2) and because it is defined from probabilities, the MIR is not easy to be calculated especially if one wants to calculate it from (chaotic) trajectories of a large complex network or data sets. The difficulties faced to estimate the MIR from dynamical systems and networks are similar to the ones faced in the calculation of the Kolmogorov-Sinai entropy, 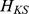 [20], (Shannon’s entropy per unit of time). Because of these difficulties, the upper bound for 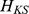 proposed by Ruelle [21] in terms of the Lyapunov exponents and valid for smooth dynamical systems (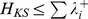, where 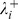 represent all the 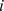 positive Lyapunov exponents) or Pesin’s equality [22] (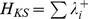) proved in Ref. [23] to be valid for the large class of systems that possess a SRB measure, became so important in the theory of dynamical systems. Our upper bound [Eq. (5)] is a result similar to the work of Ruelle, but instead we relate mutual information rate with Lyapunov exponents.

Our work is also similar to the work of Wissman-Jones-Binder [24] who have shown that upper and lower bounds for 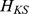 and the sum of the Lyapunov exponents can be calculated in terms of the mutual information, MI, of a trajectory. Their work, like ours, has shown a link between (conditional and joint) probabilities and a dynamical quantity, the Lyapunov exponents. We focus our attention to the relationship between MIR and Lyapunov exponents, Wissman and co-authors focus their attention in the relationship between MI and the Lyapunov exponents.

## Results

One of the main results of this work (whose derivation can be seen in Sec. Methods) is to show that, in dynamical networks or data sets with fast decay of correlation, 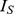 in Eq. (1) represents the amount of mutual information between 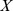 and 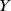 produced within a special time interval 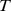, where 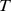 represents the time for the dynamical network (or data sets) to lose its memory from the initial state or the correlation to decay to zero. Correlation in this work is not the usual linear correlation, but a non-linear correlation defined in terms of the evolution of probabilities defined by space integrals, the quantity 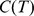 in Eq. (9). Therefore, the mutual information rate (MIR), between the dynamical variables 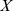 and 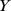 (or two data sets) can be estimated by
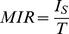
(3)


In systems that exhibit sensitivity to initial conditions, e.g. chaotic systems, predictions are only possible for times smaller than this time 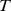. This time has other meanings. It is the expected time necessary for a set of points belonging to an 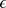-square box in 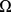 to spread over 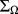 and it is of the order of the shortest Poincaré return time for a point to leave a box and return to it [25], [26]. It can be estimated by
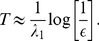
(4)where 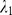 is the largest positive Lyapunov exponent measured in 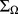. Chaotic systems can exhibit the mixing property (see Methods), and as a consequence the correlation 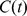 decays to zero, surely after an infinitely long time. The correlation of chaotic systems can also decay to zero for sufficiently large but finite 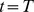 (see Information S1). 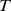 can be interpreted to be the minimum time required for a system to satisfy the conditions to be considered as mixing. Some examples of physical systems that are proved to be mixing and have exponentially fast decay of correlation are nonequilibrium steady-state [27], Lorentz gases (models of diffusive transport of light particles in a network of heavier particles) [28], and billiards [29]. An example of a “real world” physical complex system that presents exponentially fast decay of correlation is plasma turbulence [30]. We do not expect that data coming from a “real world” complex system is rigorously mixing and has an exponentially fast decay of correlation. But, we expect that the data has a sufficiently fast decay of correlation (e.g. stretched exponential decay or polynomially fast decays), implying that the system has sufficiently high sensitivity to initial conditions and as a consequence 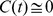, for a reasonably small and finite time 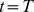


The other two main results of our work are presented in Eqs. (5) and (7), whose derivations are presented in Sec. Methods. An upper bound for the MIR is given by
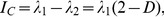
(5)where 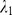 and 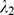 represent the largest and the second largest Lyapunov exponent measured in 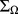, if both exponents are positive. If the 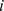-largest exponent is negative, then we set 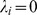. If the set 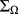 represents a periodic orbit, 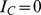, and therefore there is no information being exchanged. The quantity 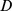 is defined as
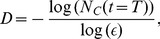
(6)where 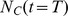 is the number of boxes that would be covered by fictitious points at time 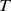. At time 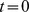, these fictitious points are confined in an 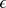-square box. They expand not only exponentially fast in both directions according to the two positive Lyapunov exponents, but expand forming a compact set, a set with no “holes”. At 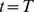, they spread over 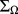.

A lower bound for the MIR is given by
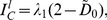
(7)where 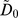 represents the capacity dimension of the set 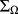


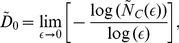
(8)where 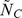 represents the number of boxes in 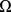 that are occupied by points of 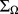.


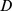
 is defined in a way similar to the capacity dimension, though it is not the capacity dimension. In fact, 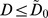, because 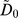 measures the change in the number of occupied boxes in 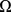 as the space resolution varies, whereas 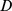 measures the relative number of boxes with a certain fixed resolution 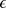 that would be occupied by the fictitious points (in 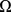) after being iterated for a time 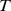. As a consequence, the empty space in 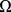 that is not occupied by 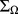 does not contribute to the calculation of 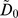, whereas it contributes to the calculation of the quantity 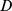. In addition, 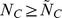 (for any 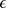), because while the fictitious points form a compact set expanding with the same ratio as the one for which the real points expand (ratio provided by the Lyapunov exponents), the real set of points 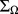 might not occupy many boxes.

## Methods

### Mixing, Correlation Decay and Invariant Measures

Denote by 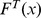 a mixing transformation that represents how a point 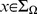 is mapped after a time 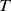 into 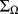, and let 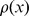 to represent the probability of finding a point of 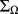 in 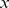 (natural invariant density). Let 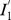 represent a region in 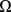. Then, 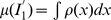, for 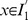 represents the probability measure of the region 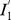. Given two square boxes 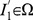 and 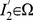, if 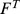 is a mixing transformation, then for a sufficiently large 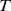, we have that the correlation defined as
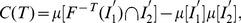
(9)decays to zero, the probability of having a point in 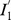 that is mapped to 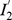 is equal to the probability of being in 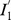 times the probability of being in 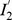. That is typically what happens in random processes.

Notice that 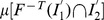 can be interpreted as a joint entropy defined by the probability of being at 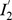 times the conditional probability (that defines elements in a transition matrix) of transferring from the set 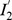 to 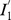.

If the measure 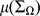 is invariant, then 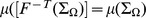. Mixing and ergodic systems produce measures that are invariant.

### Derivation of the Mutual Information Rate (MIR) in Dynamical Networks and Data Sets

We consider that the dynamical networks or data sets to be analysed present either the mixing property or have fast decay of correlations, and their probability measure is time invariant. If a system that is mixing for a time interval 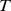 is observed (sampled) once every time interval 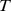, then the probabilities generated by these snapshot observations behave as if they were independent, and the system behaves as if it were a random process. This is so because if a system is mixing for a time interval 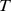, then the correlation 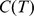 decays to zero for this time interval. For systems that have some decay of correlation, surely the correlation decays to zero after an infinite time interval. But, this time interval can also be finite, as shown in Information S1.

Consider now that we have experimental points and they are sampled once every time interval 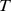. If the system is mixing, then the probability 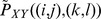 of the sampled trajectory to be in the box with coordinates 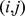 and then be iterated to the box 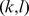 depends exclusively on the probabilities of being at the box 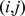, represented by 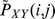, and being at the box 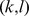, represented by 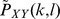.

Therefore, for the sampled trajectory, 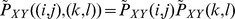. Analogously, the probability 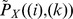 (or 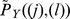) of the sampled trajectory to be in the column 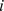 (or row 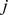) of the grid and then be iterated to the column 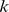 (or row 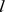) is given by 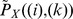  =  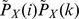 (or 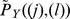  =  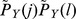).

The MIR of the experimental non-sampled trajectory points can be calculated from the mutual information of the sampled trajectory points 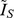 that follow itineraries of length 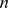:
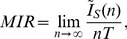
(10)


Due to the absence of correlations of the sampled trajectory points, the mutual information for these points following itineraries of length 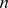 can be written as
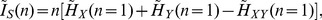
(11)where 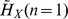  =  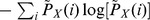, 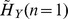  =  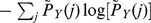, and 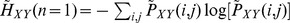, and 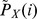, 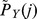, and 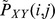 represent the probability of the sampled trajectory points to be in the column 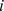 of the grid, in the row 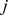 of the grid, and in the box 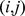 of the grid, respectively.

Due to the time invariance of the set 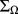 assumed to exist, the probability measure of the non-sampled trajectory is equal to the probability measure of the sampled trajectory. If a system that has a time invariant measure is observed (sampled) once every time interval 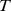, the observed set has the same natural invariant density and probability measure of the original set. As a consequence, if 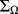 has a time invariant measure, the probabilities 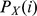, 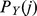, and 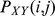 (used to calculate 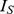) are equal to 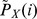, 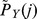, and 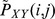.

Consequently, 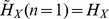, 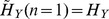, and 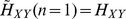, and therefore 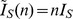. Substituting into Eq. (10), we finally arrive to 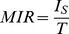 in Eq. (3), where 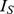 between two nodes is calculated from Eq. (1).

Therefore, in order to calculate the MIR, we need to estimate the time 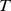 for which the correlation of the system approaches zero and the probabilities 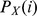, 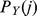, 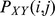 of the experimental non-sampled experimental points to fall in the column 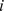 of the grid, in the row 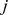 of the grid, and in the box 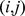 of the grid, respectively.

We demonstrate the validity of Eqs. (10) and (11) by showing that 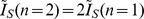, which leads to Eq. (3). For the following demonstration, 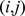 (or (k,l)) represents a box in the subspace 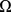 placed at coordinates 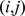, meaning a square of sides 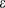 whose lower left corner point is located at 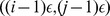. Then, 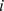 (or 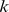) represents a column with width 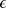 in 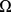 whose left side is located at 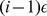 (or 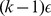) and j (or 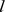) represents a row with width 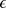 in 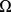 whose bottom side is located at 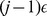 (or 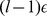).

If the system is mixing for a time 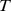, then the probability of having points in a box 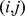 and going to another box 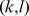, i.e., 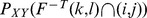 can be calculated by
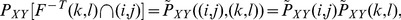
(12)


Notice that 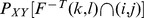 is a joint entropy that is equal to 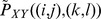, and could be written as a function of conditional probabilities: 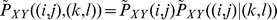, where 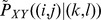 represents the conditional probability of being transferred from the box 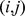 to the box 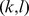.

The same can be done to calculate the probability of having points in a column 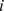 that are mapped to another column 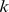, i.e. 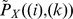, or of having points in a row 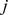 that are mapped to another row 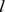, i.e. 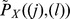. If the system is mixing for a time 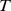, then
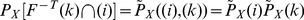
(13)and
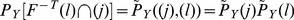
(14)for the rows. Notice that 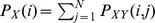 and 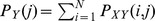.

The order-2 Mutual information of the sampled points can be calculated by.
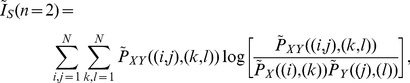
(15)where 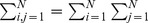. 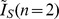 measures the MI of points that follow an itinerary of one iteration, points that are in a box and are iterated to another box. Substituting Eq. (12) in Eq. (15) we arrive at



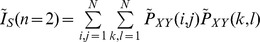


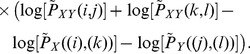
(16)


Then, substituting (13) and (14) in Eq. (16), and using the fact that 
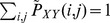
 and 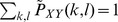, we arrive at
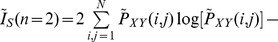


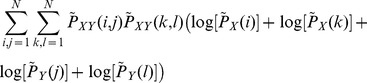
(17)


Re-organizing the terms we arrive at
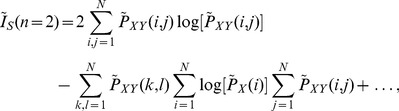
(18)where 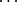 represents other terms that are similar to the term appearing in the last hand-side part of the previous equation. Using the fact that 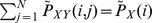, we arrive at
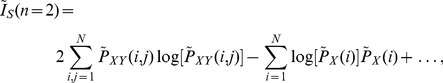
(19)which can then be written as



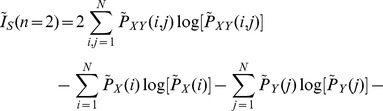


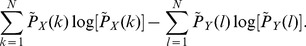
(20)


Since 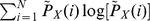  =  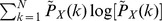 and 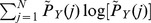  =  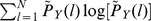, we finally arrive at that 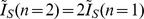. Similar calculations can be performed to state that 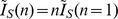. As previously discussed, 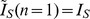, which lead us to Eq. (3).

### Derivation of an Upper (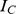) and Lower (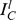) Bounds for the MIR

Consider that our attractor 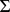 is generated by a 2d expanding system with constant Jacobian that possesses two positive Lyapunov exponents 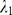 and 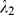, with 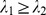. 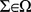. Imagine a box whose sides are oriented along the orthogonal basis used to calculate the Lyapunov exponents. Then, points inside the box spread out after a time interval 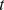 to 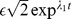 along the direction from which 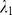 is calculated. At 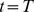, 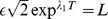, which provides 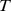 in Eq. (4), since 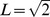. These points spread after a time interval 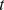 to 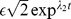 along the direction from which 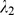 is calculated. After an interval of time 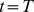, these points spread out over the set 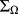. We require that for 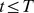, the distance between these points only increases: the system is expanding.

Imagine that at 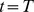, fictitious points initially in a square box occupy an area of 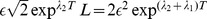. Then, the number of boxes of sides 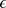 that contain fictitious points can be calculated by 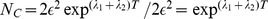. From Eq. (4), 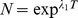, since 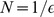.

We denote with a lower-case format, the probabilities 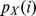, 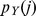, and 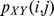 with which fictitious points occupy the grid in 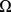. If these fictitious points spread uniformly forming a compact set whose probabilities of finding points in each fictitious box is equal, then 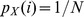 (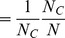), 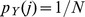, and 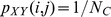. Let us denote the Shannon entropy of the probabilities 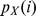, 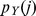 and 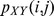 as 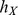, 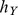, and 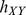, respectively. The mutual information of the fictitious trajectories after evolving a time interval 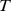 can be calculated by 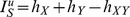. Since, 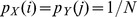 and 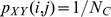, then 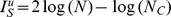. At 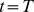, we have that 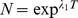 and 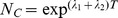, leading us to 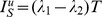. Therefore, defining, 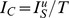, we arrive at 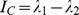.

We define 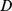 as
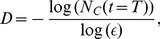
(21)where 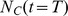 being the number of boxes that would be covered by fictitious points at time 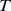. At time 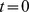, these fictitious points are confined in an -square box. They expand not only exponentially fast in both directions according to the two positive Lyapunov exponents, but expand forming a compact set, a set with no “holes”. At 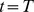, they spread over 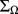.

Using 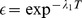 and 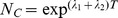 in Eq. (21), we arrive at 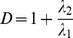, and therefore, we can write that 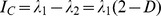, as in Eq. (5).

To calculate the maximal possible MIR, of a random independent process, we assume that the expansion of points is uniform only along the columns and rows of the grid defined in the space 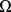, i.e., 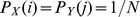, (which maximises 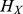 and 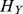), and we allow 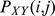 to be not uniform (minimising 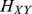) for all 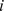 and 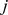, then
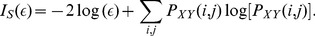
(22)


Since 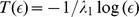, dividing 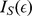 by 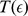, taking the limit of 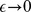, and reminding that the information dimension of the set 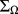 in the space 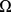 is defined as 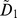 = 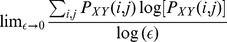, we obtain that the MIR is given by
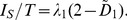
(23)


Since 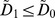 (for any value of 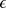), then 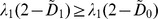, which means that a lower bound for the maximal MIR [provided by Eq. (23)] is given by 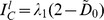, as in Eq. (7). But 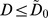 (for any value of 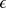), and therefore 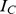 is an upper bound for 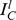.

To show why 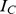 is an upper bound for the maximal possible MIR, assume that the real points 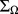 occupy the space 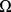 uniformly. If 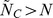, there are many boxes being occupied. It is to be expected that the probability of finding a point in a column or a row of the grid is 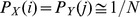, and 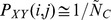. In such a case, 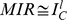, which implies that 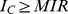. If 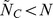, there are only few boxes being sparsely occupied. The probability of finding a point in a column or a row of the grid is 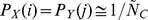, and 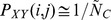. There are 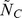 columns and rows being occupied by points in the grid. In such a case, 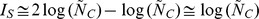. Comparing with 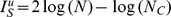, and since 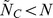 and 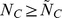, then we conclude that 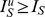, which implies that 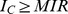.

Notice that if 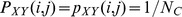 and 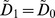, then 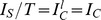.

### Expansion Rates

In order to extend our approach for the treatment of data sets coming from networks whose equations of motion are unknown, or for higher-dimensional networks and complex systems which might be neither rigorously chaotic nor fully deterministic, or for experimental data that contains noise and few sampling points, we write our bounds in terms of expansion rates defined in this work by
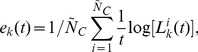
(24)where we consider 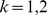. 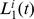 measures the largest growth rate of nearby points. In practice, it is calculated by 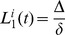, with 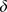 representing the largest distance between pairs of points in an 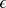-square box 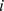 and 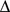 representing the largest distance between pairs of the points that were initially in the 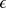-square box but have spread out for an interval of time 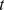. 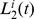 measures how an area enclosing points grows. In practice, it is calculated by 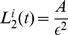, with 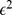 representing the area occupied by points in an 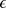-square box, and 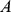 the area occupied by these points after spreading out for a time interval 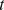. There are 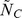 boxes occupied by points which are taken into consideration in the calculation of 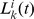. An order-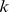 expansion rate, 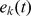, measures on average how a hypercube of dimension 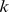 exponentially grows after an interval of time 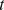. So, 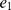 measures the largest growth rate of nearby points, a quantity closely related to the largest finite-time Lyapunov exponent [31]. And 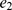 measures how an area enclosing points grows, a quantity closely related to the sum of the two largest positive Lyapunov exponents. In terms of expansion rates, Eqs. (4) and (5) read 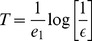 and 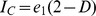, respectively, and Eqs. (6) and (7) read 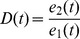 and 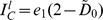, respectively.

From the way we have defined expansion rates, we expect that 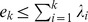. Because of the finite time interval and the finite size of the regions of points considered, regions of points that present large derivatives, contributing largely to the Lyapunov exponents, contribute less to the expansion rates. If a system has constant Jacobian, is uniformly hyperbolic, and has a constant natural measure, then 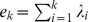.

There are many reasons for using expansion rates in the way we have defined them in order to calculate bounds for the MIR. Firstly, because they can be easily experimentally estimated whereas Lyapunov exponents demand more computational efforts. Secondly, because of the macroscopic nature of the expansion rates, they might be more appropriate to treat data coming from complex systems that contain large amounts of noise, data that have points that are not (arbitrarily) close as formally required for a proper calculation of the Lyapunov exponents. Thirdly, expansion rates can be well defined for data sets containing very few data points: the fewer points a data set contains, the larger the regions of size 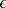 need to be and the shorter the time 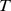 is. Finally, expansion rates are defined in a similar way to finite-time Lyapunov exponents and thus some algorithms used to calculate Lyapunov exponents can be used to calculate our defined expansion rates.

## Results and Discussion

### MIR and its Bounds in Two Coupled Chaotic Maps

To illustrate the use of our bounds, we consider the following two bidirectionally coupled maps.
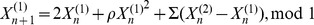


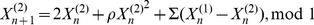
(25)where 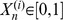. If 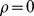, the map is piecewise-linear and quadratic, otherwise. We are interested in measuring the exchange of information between 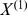 and 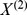. The space 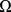 is the unit square. The Lyapunov exponents measured in the space 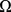 are the Lyapunov exponents of the set 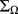 that is the chaotic attractor generated by Eqs. (25).

**Figure 1:**
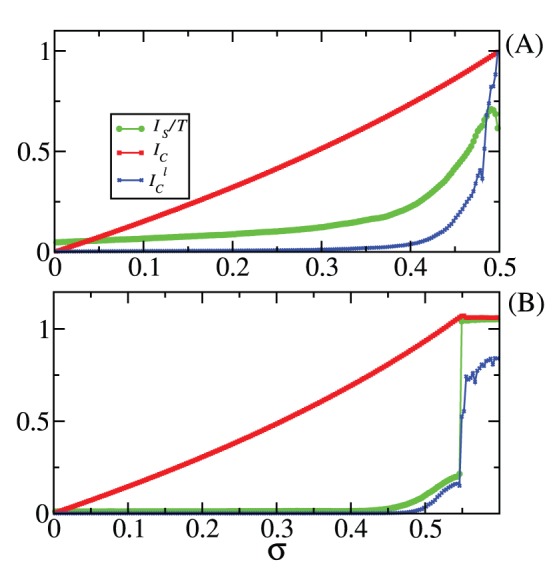
Results for two coupled maps. 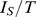 [Eq. (3)] as (green online) filled circles, 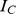 [Eq. (5)] as the (red online) thick line, and 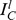 [Eq. (7)] as the (blue online) crosses. In (A) 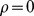 and in (B) 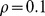. The units of 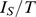, 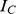, and 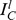 are [bits/iteration].

The quantities 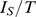, 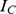, and 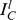 are shown in Fig. 1 as we vary 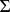 for 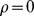 (A) and 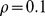 (B). We calculate 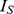 using in Eq. (1) the probabilities 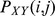 in which points from a trajectory composed of 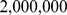 samples fall in boxes of sides 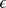 = 1/500 and the probabilities 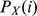 and 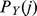 that the points visit the intervals 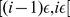 of the variable 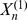 or 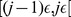 of the variable 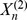, respectively, for 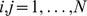. When computing 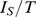, the quantity 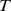 was estimated by Eq. (4). Indeed for most values of 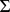, 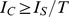 and 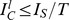.

For 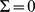 there is no coupling, and therefore the two maps are independent from each other. There is no information being exchanged. In fact, 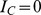 and 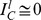 in both figures, since 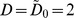, meaning that the attractor 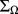 fully occupies the space 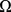. This is a remarkable property of our bounds: to identify that there is no information being exchanged when the two maps are independent. Complete synchronisation is achieved and 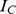 is maximal, for 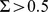 (A) and for 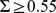 (B). A consequence of the fact that 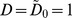, and therefore, 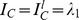. The reason is because for this situation this coupled system is simply the shift map, a map with constant natural measure; therefore 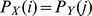 and 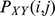 are constant for all 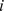 and 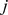. As usually happens when one estimates the mutual information by partitioning the phase space with a grid having a finite resolution and data sets possessing a finite number of points, 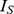 is typically larger than zero, even when there is no information being exchanged (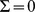). Even when there is complete synchronisation, we find non-zero off-diagonal terms in the matrix for the joint probabilities causing 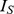 to be smaller than it should be. Due to numerical errors, 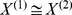, and points that should be occupying boxes with two corners exactly along a diagonal line in the subspace 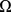 end up occupying boxes located off-diagonal and that have at least three corners off-diagonal. Due to such problems, 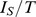 is underestimated by an amount of 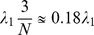, resulting in a value of approximately 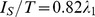, close to the value of 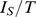 shown in Fig. 1(A), for 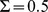. The estimation of the lower bound 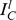 in (B) suffers from the same problems.

Our upper bound 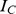 is calculated assuming that there is a fictitious dynamics expanding points (and producing probabilities) not only exponentially fast but also uniformly. The “experimental” numerical points from Eqs. (25) expand exponentially fast, but not uniformly. Most of the time the trajectory remains in 4 points: (0,0), (1,1), (1,0), (0,1). That is the main reason of why 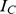 is much larger than the estimated real value of the 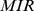, for some coupling strengths. If two nodes in a dynamical network behave in the same way the fictitious dynamics does, these nodes would be able to exchange the largest possible amount of information.

We would like to point out that one of the main advantages of calculating upper bounds for the MIR (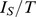) using Eq. (5) instead of actually calculating 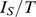 is that we can reproduce the curves for 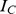 using much less number of points (1000 points) than the ones (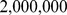) used to calculate the curve for 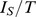. If 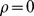, 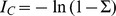 can be calculated since 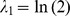 and 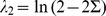.

### MIR and its Bounds in Experimental Networks of Double-Scroll Circuits

**Figure 2:**
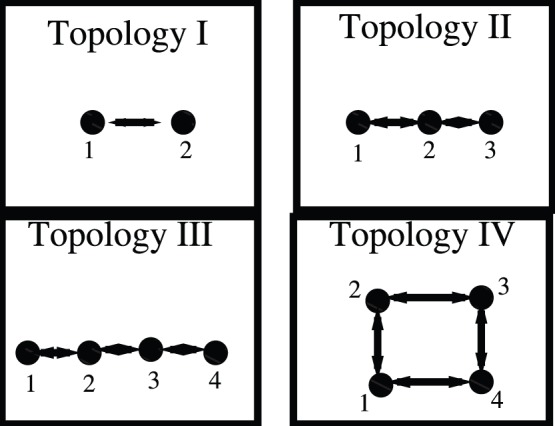
Black filled circles represent a Chua’s circuit and the numbers identify each circuit in the networks. Coupling is diffusive. We consider 4 topologies: 2 coupled Chua’s circuit (A), an array of 3 coupled circuits, an array of 4 coupled circuits, and a ring formed by 4 coupled circuits.


We illustrate our approach for the treatment of data sets using a network formed by an inductorless version of the Double-Scroll circuit [32]. We consider four networks of bidirectionally diffusively coupled circuits (see Fig. 2). Topology I in (A) represents two bidirectionally coupled circuits, Topology II in (B), three circuits coupled in an open-ended array, Topology III in (C), four circuits coupled in an open-ended array, and Topology IV in (D), coupled in an closed array. We choose two circuits in the different networks (one connection apart) and collect from each circuit a time-series of 79980 points, with a sampling rate of 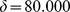 samples/s. The measured variable is the voltage across one of the circuit capacitors, which is normalised in order to make the space 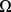 to be a square of sides 1. Such normalisation does not alter the quantities that we calculate. The following results provide the exchange of information between these two chosen circuits. The values of 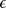 and 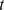 used to course-grain the space 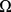 and to calculate 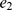 in Eq. (24) are the ones that minimise 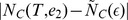 and at the same time satisfy 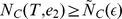, where 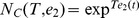 represents the number of fictitious boxes covering the set 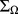 in a compact fashion, when 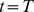. This optimisation excludes some non-significant points that make the expansion rate of fictitious points to be much larger than it should be. In other words, we require that 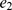 describes well the way most of the points spread. We consider that 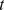 used to calculate 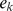 in Eq. (24) is the time points initially in an 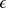-side box to become at most apart by 0.8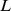. That guarantees that nearby points in 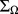 are expanding in both directions within the time interval 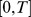. Assuming that 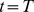 is calculated by measuring the time points initially in an 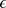-side box to be at most apart by [0.4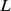, 0.8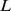] produces already similar results. If 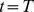 is calculated by measuring the time points become at least apart by 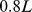, the set 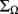 might not be only expanding. 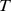 might be overestimated.

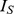
 has been estimated by the method in Ref. [33]. Since we assume that the space 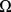 where mutual information is being measured is 2D, we will compare our results by considering in the method of Ref. [33] a 2D space formed by the two collected scalar signals. In the method of Ref. [33] the phase space is partitioned in regions that contain 30 points of the continuous trajectory. Since that these regions do not have equal areas (as it is the case for 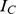 and 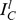), in order to estimate 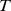 we need to imagine a box of sides 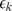, such that its area 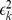 contains in average 30 points. The area occupied by the set 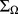 is approximately given by 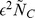, where 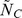 is the number of occupied boxes. Assuming that the 79980 experimental data points occupy the space 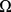 uniformly, then on average 30 points would occupy an area of 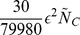. The square root of this area is the side of the imaginary box that would occupy 30 points. So, 
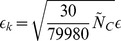
. Then, in the following, the “exact” value of the MIR will be considered to be given by 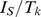, where 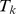 is estimated by 
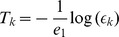
.

**Figure 3:**
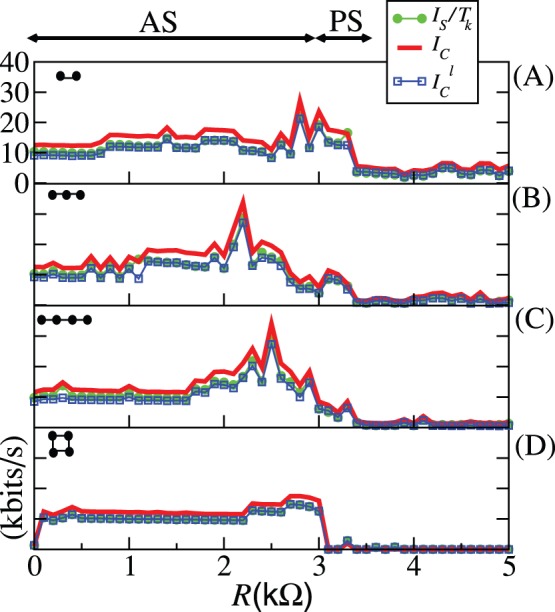
Results for experimental networks of Double-Scroll circuits. On the left-side upper corner pictograms represent how the circuits (filled circles) are bidirectionally coupled. 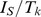 as (green online) filled circles, 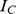 as the (red online) thick line, and 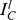 as the (blue online) squares, for a varying coupling resistance 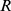. The unit of these quantities shown in these figures is (kbits/s). (A) Topology I, (B) Topology II, (C) Topology III, and (D) Topology IV. In all figures, 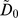 increases smoothly from 1.25 to 1.95 as 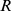 varies from 0.1k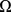 to 5k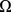. The line on the top of the figure represents the interval of resistance values responsible to induce almost synchronisation (AS) and phase synchronisation (PS).

The three main characteristics of the curves for the quantities 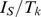, 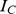, and 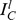 (appearing in Fig. 3) with respect to the coupling strength are that (i) as the coupling resistance becomes smaller, the coupling strength connecting the circuits becomes larger, and the level of synchronisation increases leading to an increase in 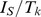, 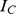, and 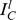, (ii) all curves are close, (iii) and as expected, for most of the resistance values, 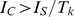 and 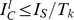. The two main synchronous phenomena appearing in these networks are almost synchronisation (AS) [34], when the circuits are almost completely synchronous, and phase synchronisation (PS) [35]. For the circuits considered in Fig. 3, AS appears for the interval 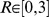 and PS appears for the interval 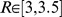. Within this region of resistance values the exchange of information between the circuits becomes large. PS was detected by using the technique from Refs. [36], [37].

### MIR and its Upper Bound in Stochastic Systems

**Figure 4:**
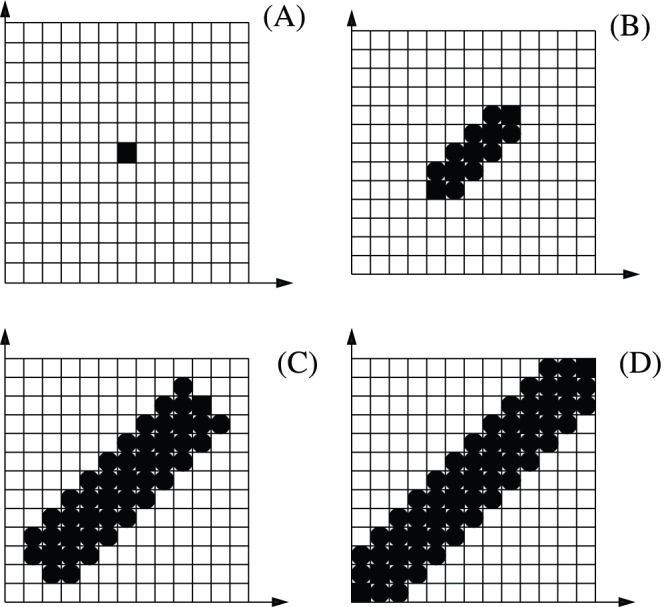
This picture is a hand-made illustration. Squares are filled as to create an image of a stochastic process whose points spread according to the given Lyapunov exponents. (A) A small box representing a set of initial conditions. After one iteration of the system, the points that leave the initial box in (A) go to 4 boxes along the diagonal line [filled squares in (B)] and 8 boxes off-diagonal (along the transverse direction) [filled circles in (B)]. At the second iteration, the points occupy other neighbouring boxes as illustrated in (C) and after an interval of time 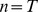 the points do not spread any longer (D).

To analytically demonstrate that the quantities 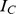 and 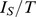 can be well calculated in stochastic systems, we consider the following stochastic dynamical toy model illustrated in Fig. 4. In it points within a small box of sides 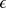 (represented by the filled square in Fig. 4(A)) located in the centre of the subspace 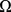 are mapped after one iteration (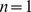, 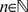) of the dynamics to 12 other neighbouring boxes. Some points remain in the initial box. The points that leave the initial box go to 4 boxes along the diagonal line and 8 boxes off-diagonal along the transverse direction. Boxes along the diagonal are represented by the filled squares in Fig. 4(B) and off-diagonal boxes by filled circles. At the second iteration (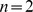), the points occupy other neighbouring boxes, as illustrated in Fig. 4(C), and at a time 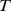 (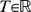) the points occupy the attractor 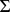 and do not spread any longer. For iterations 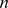 larger than 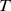, the points are somehow reinjected inside the region of the attractor. We consider that this system is completely stochastic, in the sense that no one can precisely determine the location of where an initial condition will be mapped. The only information is that points inside a smaller region are mapped to a larger region.

At the iteration 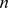, there will be 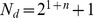 boxes occupied along the diagonal (filled squares in Fig. 4) and 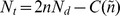 (filled circles in Fig. 4) boxes occupied off-diagonal (along the transverse direction), where 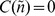 for 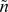 = 0, and 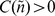 for 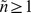 and 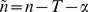. 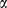 is a small number of iterations representing the time difference between the time 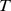 for the points in the diagonal to reach the boundary of the space 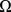 and the time for the points in the off-diagonal to reach this boundary. The border effect can be ignored when the expansion along the diagonal direction is much faster than along the transverse direction.

At the iteration 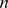, there will be 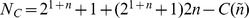 boxes occupied by points. In the following calculations we consider that 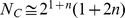. We assume that the subspace 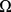 is a square whose sides have length 1, and that 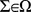, so 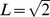. For 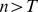, the attractor does not grow any longer along the off-diagonal direction.

The largest Lyapunov exponent or the order-1 expansion rate of this stochastic toy model can be calculated by 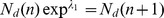, which takes us to
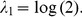
(26)


Therefore, the time 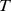, for the points to spread over the attractor 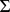, can be calculated by the time it takes for points to visit all the boxes along the diagonal. It can be calculated by 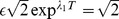, which take us to
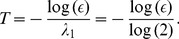
(27)


The quantity 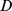 can be calculated by 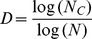, with 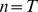. Neglecting 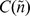 and the 1 appearing in 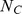 due to the initial box, we have that 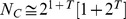. Substituting in the definition of 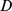, we obtain 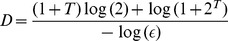. Using 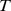 from Eq. (27), we arrive at
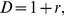
(28)where



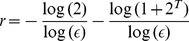
(29)Placing 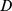 and 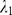 in 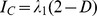, gives us
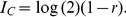
(30)


Let us now calculate 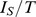. Ignoring the border effect, and assuming that the expansion of points is uniform, then 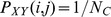 and 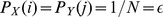. At the iteration 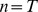, we have that 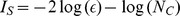. Since 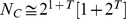, we can write that 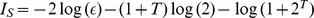. Placing 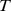 from Eq. (27) into 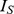 takes us to 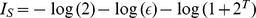. Finally, dividing 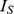 by 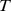, we arrive that


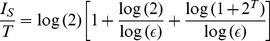


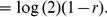
(31)


As expected from the way we have constructed this model, Eq. (31) and (30) are equal and 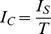.

Had we included the border effect in the calculation of 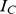, denote the value by 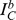, we would have obtained that 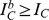, since 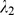 calculated considering a finite space 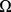 would be either smaller or equal than the value obtained by neglecting the border effect. Had we included the border effect in the calculation of 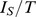, denote the value by 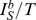, typically we would expect that the probabilities 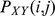 would not be constant. That is because the points that leave the subspace 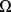 would be randomly reinjected back to 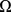. We would conclude that 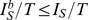. Therefore, had we included the border effect, we would have obtained that 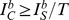.

The way we have constructed this stochastic toy model results in 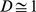. This is because the spreading of points along the diagonal direction is much faster than the spreading of points along the off-diagonal transverse direction. In other words, the second largest Lyapunov exponent, 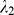, is close to zero. For stochastic toy models which produce larger 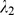, one could consider that the spreading along the transverse direction is given by 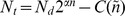, with 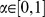.

### Expansion Rates for Noisy Data with Few Sampling Points

In terms of the order-1 expansion rate, 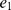, our quantities read 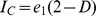, 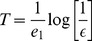, and 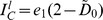. In order to show that our expansion rate can be used to calculate these quantities, we consider that the experimental system is being observed in a one-dimensional projection and points in this projection have a constant probability measure. Additive noise is assumed to be bounded with maximal amplitude 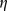, and having constant density.

Our order-1 expansion rate is defined as
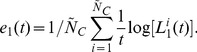
(32)where 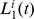 measures the largest growth rate of nearby points. Since all it matters is the largest distance between points, it can be estimated even when the experimental data set has very few data points. Since, in this example, we consider that the experimental noisy points have constant uniform probability distribution, 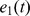 can be calculated by
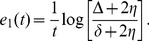
(33)where 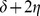 represents the largest distance between pair of experimental noisy points in an 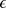-square box and 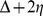 represents the largest distance between pair of the points that were initially in the 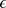-square box but have spread out for an interval of time 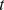. The experimental system (without noise) is responsible to make points that are at most 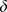 apart from each other to spread to at most to 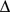 apart from each other. These points spread out exponentially fast according to the largest positive Lyapunov exponent 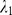 by



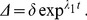
(34)Substituting Eq. (34) in (33), and expanding 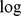 to first order, we obtain that 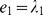, and therefore, our expansion rate can be used to estimate Lyapunov exponents.

### Conclusions

We have shown a procedure to calculate mutual information rate (MIR) between two nodes (or groups of nodes) in dynamical networks and data sets that are either mixing, or exhibit fast decay of correlations, or have sensitivity to initial conditions, and we have proposed significant upper (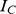) and lower (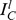) bounds for it, in terms of the Lyapunov exponents, the expansion rates, and the capacity dimension.

Since our upper bound is calculated from Lyapunov exponents or expansion rates, it can be used to estimate the MIR between data sets that have different sampling rates or experimental resolution or between systems possessing a different number of events. For example, suppose one wants to understand how much information is exchanged between two time-series, the heart beat and the level of CO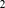 in the body. The heart is monitored by an EEG that collects data with a high-frequency, whereas the monitoring of the CO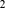 level happens in a much lower frequency. For every 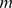 points collected from an EEG one could collect 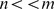 points in the monitoring of the CO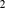 level. Assuming that the higher-frequency variable (the heart beat) is the one that contributes mostly for the sensibility to the initial conditions, then the larger expansion rate (or Lyapunov exponent) can be well estimated only using this variable. The second largest expansion rate (or Lyapunov exponent) can be estimated by the composed subspace formed by these two measurements, but only the measurements taken simultaneously would be considered. Therefore, the estimation of the second largest expansion rate would have to be done using less points than the estimation used to obtain the largest. In the calculation of the second largest expansion rate, it is necessary to know the largest exponent. If the largest is correctly estimated, then the chances we make a good estimation of the second largest increases, even when only a few points are considered. With the two largest expansion rates, one can estimate 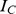, the upper bound for the MIR.

Additionally, Lyapunov exponents can be accurately calculated even when data sets are corrupted by noise of large amplitude (observational additive noise) [38], [39] or when the system generating the data suffers from parameter alterations (“experimental drift”) [40]. Our bounds link information (the MIR) and the dynamical behaviour of the system being observed with synchronisation, since the more synchronous two nodes are, the smaller 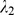 and 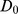 will be. This link can be of great help in establishing whether two nodes in a dynamical network or in a complex system not only exchange information but also have linear or non-linear interdependences, since the approaches to measure the level of synchronisation between two systems are reasonably well known and are been widely used. If variables are synchronous in a time-lag fashion [35], it was shown in Ref. [16] that the MIR is independent of the delay between the two processes. The upper bound for the MIR could be calculated by measuring the Lyapunov exponents of the network (see Information S1), which are also invariant to time-delays between the variables.

If the MIR and its upper bounds are calculated from an “attractor” that is not an asymptotic limiting set but rather a transient trajectory, these values should typically differ from the values obtained when the "attractor" is an asymptotic limiting set. The dynamical quantities calculated, e.g., the Lyapunov exponents, expansion rates, and the fractal dimension should be interpreted as finite time quantities.

In our calculations, we have considered that the correlation of the system decays to approximately zero after a finite time 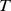. If after this time interval the correlation does not decay to zero, we expect that 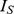 will be overestimated, leading to an overestimated value for the MIR. That is so because the probabilities used to calculate 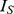 will be considered to have been generated by a random system with uncorrelated variables, which is not true. However, by construction, the upper bound 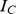 is larger than the overestimated MIR.

## Supporting Information

Information S1 (PDF)
