# Supplementary material for: Mutual Information Rate and Bounds for It
Source: PLoS One. 2012 Oct 24;7(10):e46745. doi: 10.1371/journal.pone.0046745 (PMC3480398; doi:10.1371/journal.pone.0046745)
Supplement: Information S1 — (PDF) [file pone.0046745.s001.pdf]

I. SUPPLEMENTARY INFORMATION

A. Decay of correlation and First Poincaré Returns

As rigorously shown in [1], the decay with time of the correlation, $C(t)$, is proportional to the decay with time of the density of the first Poincaré recurrences, $\rho(t, \epsilon)$, which measures the probability with which a trajectory returns to an ϵ-interval after t iterations. Therefore, if $\rho(t, \epsilon)$ decays with t, for example exponentially fast, $C(t)$ will decay with t exponentially fast, as well. The relationship between $C(t)$ and $\rho(t)$ can be simply understood in chaotic systems with one expanding direction (one positive Lyapunov exponent). As shown in [2], the "local" decay of correlation (measured in the ϵ-interval) is given by $C(t, \epsilon) \leq \mu(\epsilon)\rho(t, \epsilon) - \mu(\epsilon)^2$, where $\mu(\epsilon)$ is the probability measure of a chaotic trajectory to visit the ϵ-interval. Consider the shift map $x_{n+1} = 2x_n, \text{mod } 1$. For this map, $\mu(\epsilon) = \epsilon$ and there are an infinite number of possible intervals that makes $C(t, \epsilon) = 0$, for a finite t. These intervals are the cells of a Markov partition. As recently demonstrated by [P. Pinto, I. Labouriau, M. S. Baptista], in piecewise-linear systems as the shift map, if ϵ is a cell in an order-t Markov partition and $\rho(t, \epsilon) > 0$, then $\rho(t, \epsilon) = 2^{-t}$ and by the way a Markov partition is constructed we have that $\epsilon = 2^{-t}$. Since that $\epsilon = \mu(\epsilon) = 2^{-t}$, we arrive at that $C(t, \epsilon) \leq 0$, for a special finite time t. Notice that $\epsilon = 2^{-t}$ can be rewritten as $-\ln(\epsilon) = t \ln(2)$. Since for this map, the largest Lyapunov exponent is equal to $\lambda_1 = \ln(2)$, then $t = -\frac{1}{\lambda_1} \ln(\epsilon)$, which is exactly equal to the quantity T, the time interval responsible to make the system to lose its memory from the initial condition and that can be calculated by the time that makes points inside an initial ϵ-interval to spread over the whole phase space, in this case $[0, 1]$.

B. I_C, and I_C^l in larger networks and higher-dimensional subspaces Σ_Ω

Imagine a network formed by K coupled oscillators. Uncoupled, each oscillator possesses a certain amount of positive Lyapunov exponents, one zero, and the others are negative. Each oscillator has dimension d. Assume that the only information available from the network are two Q dimensional measurements, or a scalar signal that is reconstructed to a Q-dimensional embedding space. So, the subspace Σ_Ω has dimension $2Q$, and each subspace of a node (or group of nodes) has dimension Q. To be consistent with our previous equations, we assume that we measure $M_\Omega = 2Q$ positive Lyapunov exponents on the projection Σ_Ω. If $M_\Omega \neq 2Q$, then in the following equations $2Q$ should be replaced by M_Ω, naturally assuming that $M_\Omega \leq 2Q$.

In analogy with the derivation of I_C and I_C^l in a bidimensional projection, we assume that if the spreading of initial conditions is uniform in the subspace Ω. Then, $P_X(i) = \frac{1}{N^Q}$ represents the probability of finding trajectory points in Q-dimensional space of one node (or a group of nodes) and $P_{XY}(i, j) = \frac{1}{N_C}$ represents the probabilities of finding trajectory points in the $2Q$-dimensional composed subspace constructed by two nodes (or two groups of nodes) in the subspace Ω. Additionally, we consider that the hypothetical number of occupied boxes N_C will be given by $N_C(T) = \exp^{T(\sum_{i=1}^{2Q} \lambda_i)}$. Then, we have that $T = 1/\lambda_1 \log(N)$, which lead us to

$$I_C = \lambda_1(2Q - D). \quad (1)$$

Similarly to the way we have derived I_C^l in a bidimensional projection, if Σ_Ω has more than 2 positive Lyapunov exponents, then

$$I_C^l = \lambda_1(2Q - \tilde{D}_0). \quad (2)$$

To write Eq. (1) in terms of the positive Lyapunov exponents, we first extend the calculation of the quantity D to higher-dimensional subspaces that have dimensionality 2Q,

$$D = 1 + \sum_{i=2}^{2Q} \frac{\lambda_i}{\lambda_1}, \quad (3)$$

where $\lambda_1 \geq \lambda_2 \geq \lambda_3 \ldots \geq \lambda_{2Q}$ are the Lyapunov exponents measured on the subspace Ω. To derive this equation we only consider that the hypothetical number of occupied boxes N_C is given by $N_C(T) = \exp^{T(\sum_{i=2}^{2Q} \lambda_i)}$.

We then substitute D as a function of these exponents (Eq. (3)) in Eq. (1). We arrive at

$$I_C = (2Q - 1)\lambda_1 - \sum_{i=2}^{2Q} \lambda_i. \quad (4)$$

C. I_C as a function of the positive Lyapunov exponents of the network

Consider a network whose attractor Σ possesses M positive Lyapunov exponents, denoted by $\tilde{\lambda}_i$, $i = 1, \ldots, M$, and that can be calculated either directly or via the reconstructed attractor. For a typical subspace Ω, λ_1 measured on Ω is equal to the largest Lyapunov exponent of the network. Just for the sake of simplicity, assume that the nodes in the network are sufficiently well connected so that in a typical measurement with a finite number of observations this property holds, i.e., $\tilde{\lambda}_1 = \lambda_1$. But, if measurements provide that $\tilde{\lambda}_1 >> \lambda_1$, the next arguments apply as well, if one replaces $\tilde{\lambda}_1$ appearing in the further calculations by the smallest Lyapunov exponent, say, $\tilde{\lambda}_k$, of the network that is still larger than λ_1, and then, substitute $\tilde{\lambda}_2$ by $\tilde{\lambda}_{k+1}$, and so on. As before, consider that $M_\Omega = 2Q$.

Then, for an arbitrary subspace Ω, $\sum_{i=2}^{2Q} \lambda_i \leq \sum_{i=2}^{2Q} \tilde{\lambda}_i$, since a projection cannot make the Lyapunov exponents larger, but only smaller or equal.

Defining

$$\tilde{I}_C = (2Q-1)\lambda_1 - \sum_{i=2}^{2Q} \tilde{\lambda}_i. \quad (5)$$

Since $\sum_{i=2}^{2Q} \lambda_i \leq \sum_{i=2}^{2Q} \tilde{\lambda}_i$, it is easy to see that

$$\tilde{I}_C \leq I_C. \quad (6)$$

So, I_C, measured on the subspace Σ_Ω and a function of the $2Q$ largest positive Lyapunov exponents measured in Σ_Ω, is an upper bound for $\tilde{I}_C$, a quantity defined by the $2Q$ largest positive Lyapunov exponents of the attractor Σ of the network. Therefore, if the Lyapunov exponents of a network are know, the quantity $\tilde{I}_C$ can be used as a way to estimate how much is the MIR between two measurements of this network, measurements that form the subspace Ω.

Notice that I_C depends on the projection chosen (the subspace Ω) and on its dimension, whereas $\tilde{I}_C$ depends on the dimension of the subspace Σ_Ω (the number 2Q of positive Lyapunov exponents). The same happens for the mutual information between random variables that depend on the projection considered.

The Lyapunov exponents of the network, and consequently $\tilde{I}_C$, can be obtained from the reconstructed attractor of a single time-series measure. Hence, the reconstructed attractor can be used to estimate I_C.

Equation (5) is important because it allows us to obtain an estimation for the value of I_C analytically. As an example, imagine the following network of coupled maps with a constant Jacobian

$$X_{n+1}^{(i)} = 2X_n^{(i)} + \sigma \sum_{j=1}^{K} \mathbf{A}_{ij}(X_n^{(j)} - X_n^{(i)}), \text{mod } 1, \quad (7)$$

where $X \in [0,1]$ and $\mathbf{A}$ represents the connecting adjacent matrix. If node j connects to node i, then $\mathbf{A}_{ij} = 1$, and 0 otherwise.

Assume that the nodes are connected all-to-all. Then, the K positive Lyapunov exponents of this network are: $\tilde{\lambda}_1 = \log(2)$ and $\tilde{\lambda}_i = \log 2[1+\sigma]$, with $i = 2, K$. Assume also that the subspace Ω has dimension $2Q$ and that $2Q$ positive Lyapunov exponents are observed in this space and that $\tilde{\lambda}_1 = \lambda_1$. Substituting these Lyapunov exponents in Eq. (5), we arrive at

$$\tilde{I}_C = (2Q-1)\log(1+\sigma). \quad (8)$$

We conclude that there are two ways for $\tilde{I}_C$ to increase. Either one considers larger measurable subspaces Ω or one increases the coupling between the nodes. This suggests that the larger the coupling strength is the more information is exchanged between groups of nodes.

For arbitrary topologies, one can also derive analytical formulas for $\tilde{I}_C$ in this network, since $\tilde{\lambda}_i$ for $i > 2$ can be calculated from $\tilde{\lambda}_2$ [3]. One arrives at

$$\tilde{\lambda}_i(\omega_i \sigma/2) = \tilde{\lambda}_2(\sigma), \quad (9)$$

where ω_i is the ith largest eigenvalue (in absolute value) of the Laplacian matrix $\mathbf{L}_{ij} = \mathbf{A}_{ij} + \mathbb{I} \sum_j \mathbf{A}_{ij}$.

[1] Young LS (1999) Israel Journal of Mathematics 110: 153-188.

[2] Baptista MS, Maranhão DM, Sartorelli JC (2009) Chaos 19: 043115-1-043115-10.

[3] Baptista MS, Kakmeni FM, Magno GL, Hussein MS (2011) Phys. Lett. A 375: 1309-1318.
